# Vision-based tracking system for augmented reality to localize recurrent laryngeal nerve during robotic thyroid surgery

**DOI:** 10.1038/s41598-020-65439-6

**Published:** 2020-05-21

**Authors:** Dongheon Lee, Hyeong Won Yu, Seunglee Kim, Jin Yoon, Keunchul Lee, Young Jun Chai, June Young Choi, Hyoun-Joong Kong, Kyu Eun Lee, Hwan Seong Cho, Hee Chan Kim

**Affiliations:** 1 Interdisciplinary Program, Bioengineering Major, Graduate School, Seoul National University, Seoul, Korea; 2 Department of Surgery, Seoul National University Bundang Hospital, Seongnam-si, South Korea; 3 MAXST, Seoul, South Korea; 4 Department of Surgery, Seoul National University Boramae Medical Center, Seoul, South Korea; 5 Department of Biomedical Engineering, Chungnam National University College of Medicine, Daejeon, Korea; 6 Department of Surgery, Seoul National University Hospital and College of Medicine, Seoul, South Korea; 7 Department of Orthopaedic Surgery, Seoul National University Bundang Hospital, Seongnam-si, South Korea; 8 Department of Biomedical Engineering, College of Medicine and Institute of Medical and Biological Engineering, Medical Research Center, Seoul National University, Seoul, Korea

**Keywords:** Biomedical engineering, Nervous system

## Abstract

We adopted a vision-based tracking system for augmented reality (AR), and evaluated whether it helped surgeons to localize the recurrent laryngeal nerve (RLN) during robotic thyroid surgery. We constructed an AR image of the trachea, common carotid artery, and RLN using CT images. During surgery, an AR image of the trachea and common carotid artery were overlaid on the physical structures after they were exposed. The vision-based tracking system was activated so that the AR image of the RLN followed the camera movement. After identifying the RLN, the distance between the AR image of the RLN and the actual RLN was measured. Eleven RLNs (9 right, 4 left) were tested. The mean distance between the RLN AR image and the actual RLN was 1.9 ± 1.5 mm (range 0.5 to 3.7). RLN localization using AR and vision-based tracking system was successfully applied during robotic thyroidectomy. There were no cases of RLN palsy. This technique may allow surgeons to identify hidden anatomical structures during robotic surgery.

## Introduction

Robotic thyroid surgery has become popular over the past decade because it does not leave a scar on the neck, unlike traditional open surgery^1^. Although robotic thyroid surgery offers a three-dimensional magnified operative view, tremor filtering system, and endo-wrist movement, the surgeon cannot use tactile sense, and identifying covered anatomical structures is difficult, compared to open surgery^2^.

Injury to the RLN is a major complication of thyroid surgery that can result in vocal cord palsy, aspiration, and poor quality of life^3^. Thus, during thyroid surgery, preservation of recurrent laryngeal nerve (RLN) function is very important. However, because the RLN is located posteriorly to the thyroid glands, it is difficult to identify, making it susceptible to injury during exploration. During robotic thyroid surgery, it is challenging to preserve RLN function because the surgeon cannot use tactile sense.

Augmented reality (AR) in surgery is a new technique that enables virtual images of the organs to be overlaid onto the actual organs during surgery^4–6^. Currently, application of AR in surgery is unpopular because it is a cumbersome process: AR images do not follow the robot’s camera movement, so they must be manually relocated according to camera movement. To overcome this limitation, we applied a vision-based tracking system that allows the AR image to move in line with the actual organ, following camera movement. We evaluated whether this system aids the surgeon to localize the RLN during robotic thyroid surgery.

## Results

In the pilot study, seven RLNs (4 right and 3 left) from six patients were used to measure the distance between the actual RLN and the trachea (Table 1). The mean distance was 7.5 mm.

**Table 1:** Pilot study data.

Patient no.	Sex	Age	Side	Distance between trachea and RLN, mm
1	M	46	Lt	8.2
2	M	38	Rt	7.8
3	F	33	Rt	6.6
4	F	60	Lt	8.0
			Rt	9.4
5	F	42	Rt	5.0
6	M	33	Lt	7.2
Mean ± SD				7.5

RLN; recurrent laryngeal nerve.

In this prospective study, nine patients were enrolled and 11 RLNs (9 right and 4 left) were tested (Table 2). After activation of the vision-based tracking system, AR images moved in line with the actual organs (Supplementary Video). The distance between the AR image of the RLN and the actual RLN was 1.9 ± 1.5 mm (range 0.5 to 3.7; Table 3). There were no RLN palsy cases on postoperative laryngoscopic examination.

**Table 2:** Demographics of the study patients.

Patient no.	Sex	Age	Side	Diagnosis	Tumor size, cm
1	M	29	Rt	PTC	0.7
2	F	33	Rt	PTC	2.2
3	F	31	Rt	PTC	0.9
4	F	49	Rt	PTC	
			Lt	PTC	1.0
5	M	43	Rt	PTC	0.8
6	F	29	Lt	PTC	0.8
7	F	27	Rt	PTC	0.9
			Lt	PTC	0.5
8	F	59	Lt	PTC	3.7
9	F	54	Rt	PTC	0.4

PTC; papillary thyroid carcinoma.

**Table 3:** Distance between the AR image of the RLN and actual RLN.

Patient no.	Surgical site	RMSE, mm
1	Rt	0.9
2	Rt	0.5
3	Rt	0.4
4	Rt	3.7
	Lt	0.5
5	Rt	0.5
6	Lt	3.4
7	Rt	1.9
	Lt	4.7
8	Lt	1.3
9	Rt	3.1
Mean ± SD		1.9 ± 1.5

AR; augmented reality, RLN; recurrent laryngeal nerve, RMSE, root mean squared error.

## Discussion

Identification of hidden structures is more challenging during robotic surgery than open surgery because of the loss of tactile feedback and difficulties with hand-eye coordination with instruments^7^. Identifying the RLN, the most time-consuming step during thyroid surgery, is more challenging during robotic thyroid surgery compared to open surgery. AR is a technology that superimposes images of objects onto physical objects^8,9^. AR approaches are employed in surgery for hidden or deeply located tumors such as parathyroid, liver, or brain tumors^10–12^. The technology is also applied in robotic surgery^7^. However, the AR image is unable to follow camera movement during surgical procedures, thus it requires continuous manual overlaying or the use of additional instruments for image localization^1,13,14^.

In this study, we developed a semi-automatic registration method to enable an AR image to be automatically overlaid on the actual organs followed by manual fine-tuning. Constructing AR images of the common carotid artery, trachea, and RLN took about 30 minutes in total. For automatic overlaying, the color and axis of the trachea were used as a landmark. The actual tracheal region was segmented using the thresholding method which is a technique used to detect color difference. The angles of the long and short axes of the segmented trachea regions were determined. The AR image of the trachea was overlaid onto the actual tracheal region at the predetermined angle. To enable the AR of the RLN to follow camera movement, we applied simultaneous localization and mapping (SLAM) technology^15,16^.

SLAM technology constructs a 3D map of the surrounding environment almost in real-time while simultaneously tracking an object’s location^17–19^. The proposed tracking system is also capable of detecting respiratory movement, as shown in Supplementary Video. This technology does not require training with images and is widely used across a range of fields including navigation, robotic mapping, odometry for virtual reality, and AR^20–23^. In this study, vision-based tracking was performed in three steps: (1) SLAM application on the physical surgical image, (2) semi-automatic organ overlay, and (3) vision-based organ tracking for AR images to follow camera movement in real-time.

There are limitations to this study. We were unable to display AR on the surgeon’s binocular monitor (on the robot) because integration of AR on the robotic monitor is prohibited by the equipment’s license. Instead, we used a separate monitor to display and manipulate the AR. Integration will be possible in the future when manufactures make surgical robots accessible by AR software. Another limitation of this technique is that the current system does not compensate for tissue deformation. We constructed AR images for the non-deformable structure (trachea) and the deformable structures (common carotid artery and RLN). However, we did not use a deformable registration method in this study for two reasons: First, the common carotid artery and RLN can be regarded as fixed organs because their position is relatively fixed, although their nature is deformable. Second, a minor inaccuracy in the location of the RLN is not clinically significant and was acceptable to the surgeon. The role of AR in this study was not to predict the exact location of the RLN, but to suggest the probable location of the RLN and help the surgeon to explore the area around the RLN.

In summary, we developed a vision-based tracking system for AR using SLAM technology and showed that RLN can be successfully localized during robotic thyroidectomy. This technique may be helpful for surgeons to identify hidden anatomical structures during robotic surgery.

## Methods

### Patients

This prospective study was approved by the Institutional Review Boards of SMG-SNU Boramae Medical Center and Seoul National University Bundang Hospital (IRB No. L-2018-377 and B-1903/531-403). All methods were carried out in accordance with regulations and institutional guidelines. This study was performed in patients who underwent bilateral axillo-breast approach robotic thyroid surgery due to papillary thyroid carcinoma tumor from March to April 2019. Patients were counseled about using AR and vision-based tracking system and informed consent was provided by each patient before surgery. The robot model used in this study was the Xi model of the da Vinci Surgical System (Intuitive Surgical Inc., Sunnyvale, CA, USA).

### AR image construction

Based on the DICOM files, AR images of the structures of interest (common carotid artery, trachea, RLN) were constructed using open source software Seg3D (v.2.4.3, National Institutes of Health Center for Integrative Biomedical Computing at the University of Utah Scientific Computing and Imaging Institute, Salt Lake City, UT, USA).

**Figure 1:**
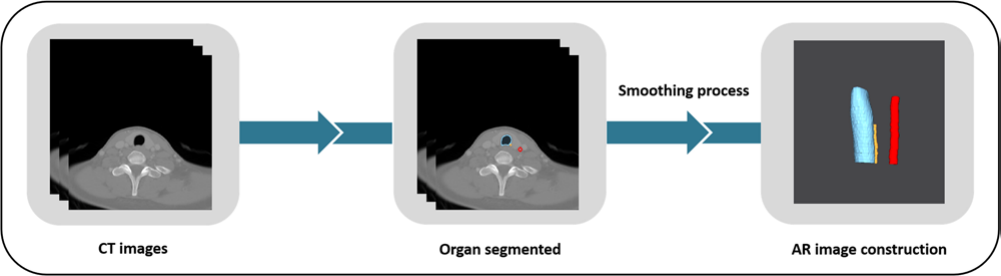
Process of augmented reality image construction trachea, common carotid artery, and recurrent laryngeal nerve.

Figure 1 demonstrates AR image construction. A thresholding method was used for the trachea AR image (threshold value ranged −2,650 to −240 HU), and AR images of the common carotid artery and the RLN were manually segmented. 3D volume rendering was performed using a marching cubes algorithm through segmented section images^24^. The surface of the AR image was post-processed with smoothing using MeshMixer (v.3.5.474, Autodesk Inc., San Rafael, CA, USA).

### Study protocol

In the pilot study, AR images of the trachea and common carotid artery were constructed based on CT images. The images were overlaid on the actual organs during surgery. After the RLN was identified, the distance between the actual RLN and the AR image of the trachea was measured.

In the prospective study, AR images of the trachea, common carotid artery, and the RLN were constructed using CT images. The AR image of the RLN was positioned to the side of the trachea at the distance determined by the pilot study. During robotic thyroid surgery, the AR images of the trachea and common carotid artery were overlaid on the actual structures after they were exposed. The vision-based tracking system was activated so that the AR image of the RLN followed the camera movement. After identifying the actual RLN, the distance between the AR image of the RLN and the actual RLN was measured.

### Hardware of tracking system in robotic surgery

**Figure 2:**
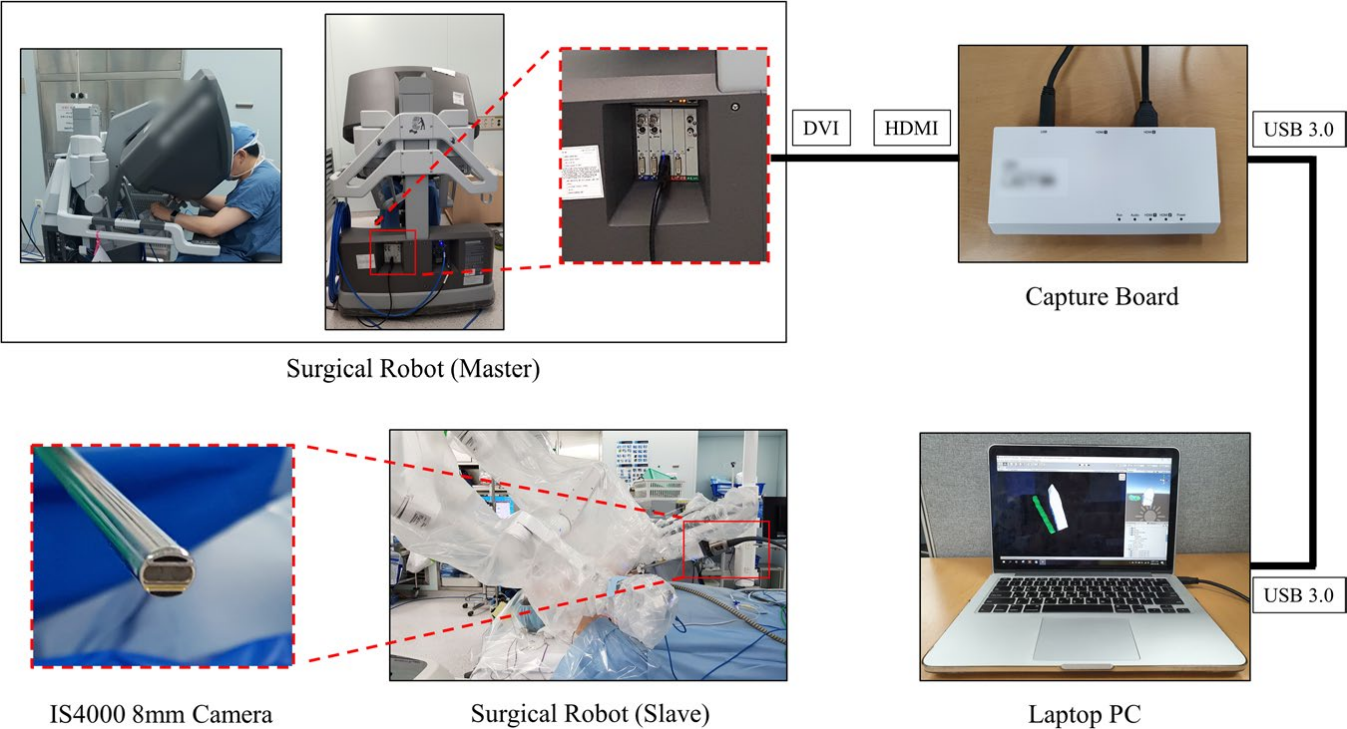
Hardware of tracking system used in robotic surgery. The surgical robot screen is branched out and connected to a laptop computer operating a vision-based tracking system.

Figure 2 shows the hardware of the tracking system used during robotic surgery. The AR screen is branched from the screen of the master surgical robot using a capture board, and connected to a laptop computer running a vision-based tracking system. We used the IS4000 8 mm camera provided with the Xi model of the da Vinci Surgical System. It is a 3D camera consisting of two lenses, but only a single image capture by a 2D monocular camera was used in this study. The camera tip angles was 30 degrees, and the working distance range was 20 mm to 40 mm. The field of view was 70 to 80 degrees, and the focal length was 8 mm. The video specification was 1920 × 1080 resolution, and 30 fps.

### Procedures of AR application using vision-based tracking system

**Figure 3:**
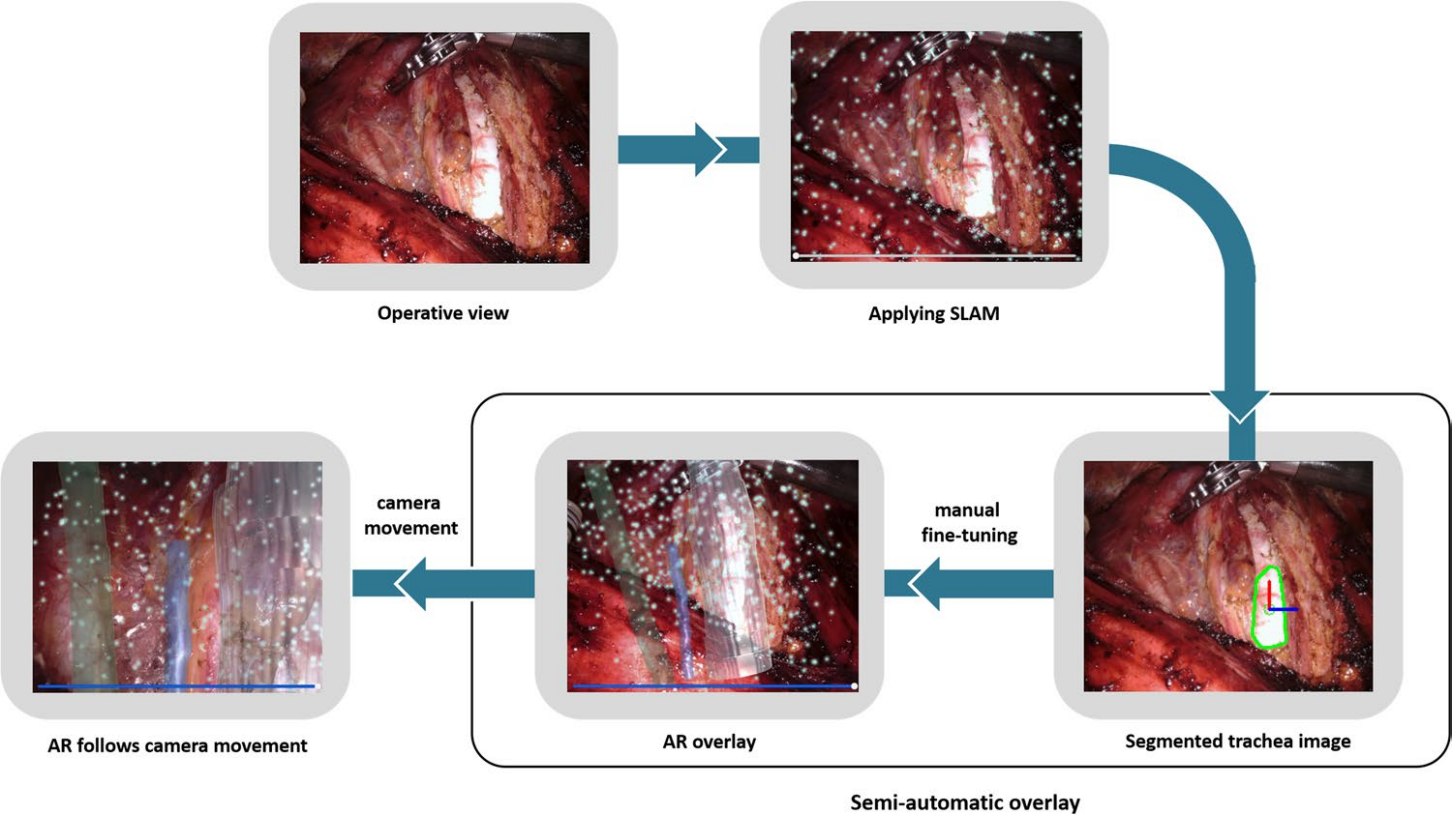
Process of AR application on the operative image using vision-based tracking system.

**Figure 4:**
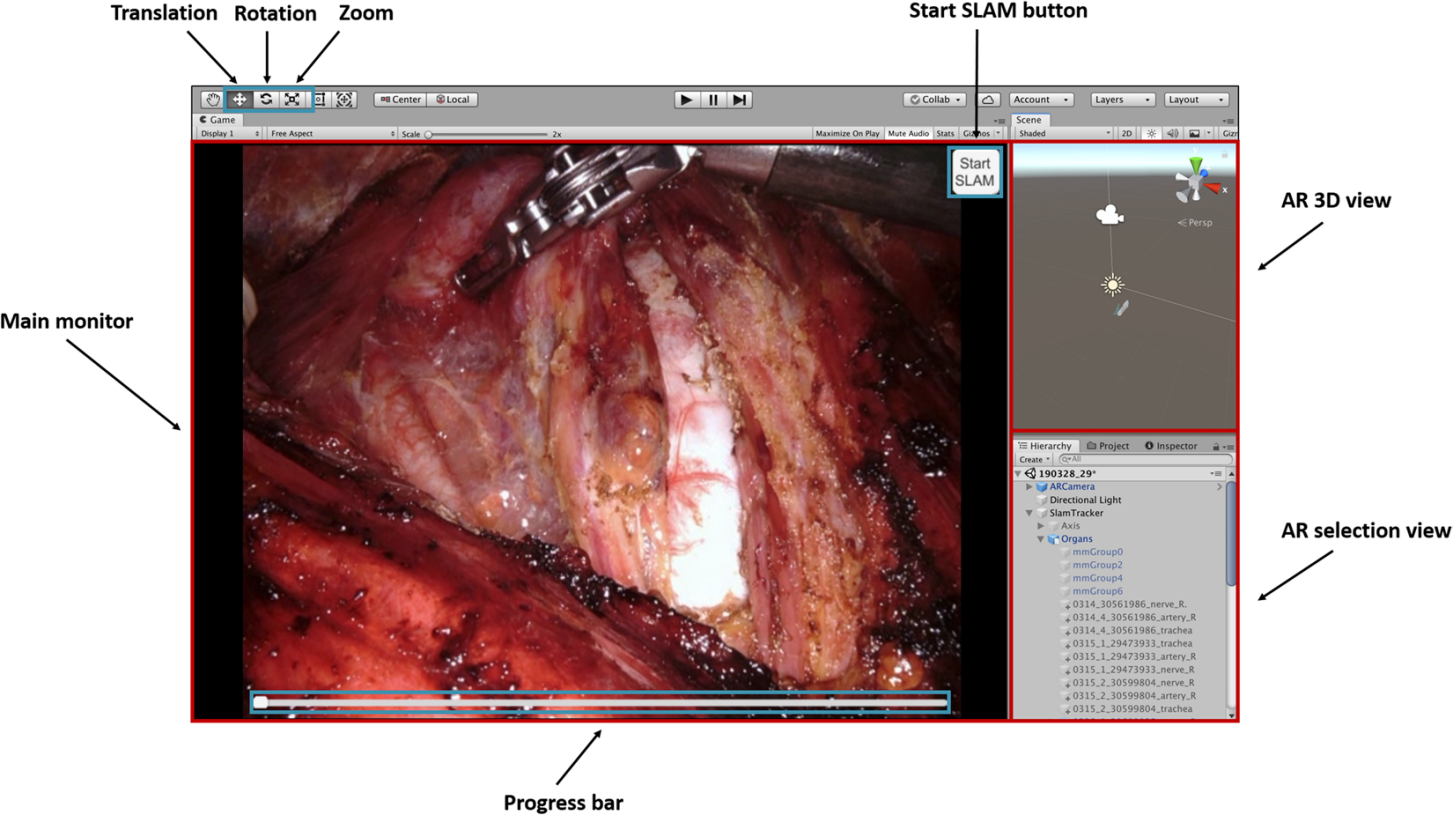
Development of an environment for augmented reality image tracking system. The system is controlled by mouse and provides translation, rotation, and zoom function. The progress bar at the bottom of the screen gives information about the sufficiency of recognized feature points for map generation.

Figure 3 demonstrates the process of AR application and vision-based organ tracking. After exposing the trachea and common carotid artery, SLAM technology was applied to create a 3D map of the operative field with the ability to hold AR images after overlay. We used a 3D-2D registration method to apply a constructed 3D AR image to a 2D image obtained through a monocular surgical camera. AR images of the trachea and common carotid artery were overlaid semi-automatically. Color and axis of the segmented tracheal region were used for initial automatic overlay, and the location of the AR image was fine-tuned manually through translation (x, y, z), rotation (roll, pitch, yaw), and zoom (in, out) using a mouse control. Following the development of an environment for the tracking system to operate within, we developed the tracking system using OpenCV (v2.3.8) for Unity software (v.2018.3.7 f1, Unity Software Inc, San Francisco, CA, USA), as shown in Fig. 4.

SLAM technology, using MAXST AR SDK software (v4.1.4, MAXST, Seoul, Korea), was applied to create a 3D map of the operative field with the ability to hold AR images after semi-automatic overlay. This technology has the ability to track an object or a space without any prior knowledge in real-time using only visual information from a camera^25,26^. SLAM achieves 3D point mapping via stereo matching using motion parallax between the first and subsequent camera images. The points are tracked and 6 degrees of freedom (6-DoF) camera pose is estimated. The tracking algorithm first calculates a rough relative pose between contiguous images by frame-to-frame matching, and projects the 3D points onto the current image by the pose. Then, the algorithm searches their corresponding images to gather matched pairs between 3D and 2D^27^. The final pose is optimized iteratively from the pairs^28^. If the image includes an unknown scene, some feature points are extracted from the unknown scene, triangulated by epipolar geometry, and added to the 3D point map^29^.

To apply SALM in this study, the surgeon moved the robotic camera continuously for about 5-10 seconds at various angles to create an initial 3D point map. Then, the vision-based tracking system was applied for AR images to follow camera movement in line with the actual organs, in real-time. This method employs a coarse-to-fine strategy to cope with motion blur or fast movement of a camera and efficient second-order minimization^30^ (ESM) algorithm for precise tracking. When a camera moves fast, the correspondence search often fails due to the large search range. To overcome this problem, the input image is configured into a four-level image pyramid and the smallest image is utilized in the coarse step where a rotation of only 3-DoF pose is calculated quickly between contiguous images via the ESM algorithm. The rough 3-DoF pose reduces the correspondence search range in the fine step, where the small image patches tagged with 3D coordinate are searched on the input image via the ESM algorithm. The ESM algorithm matches quickly between the two images and shows sub-pixel matching accuracy. The software used in this study is free for public use, except for MAXST AR SDK which was used to overlay AR images onto the robotic surgery view. Clinicians without knowledge of bioengineering can use the software.

### Distance measurement

**Figure 5:**
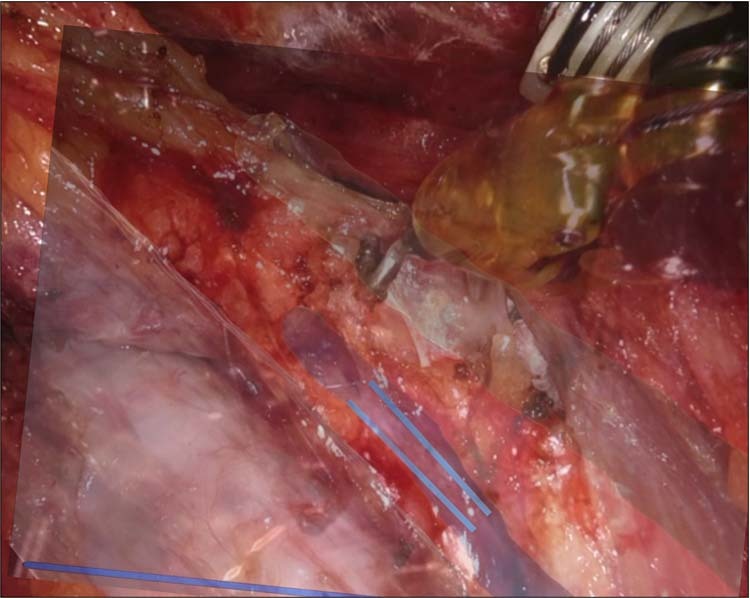
Measurement of the distance between the augmented reality image of the recurrent laryngeal nerve (blue) and actual recurrent laryngeal nerve (light blue double line). augmented reality images of trachea (white) and common carotid artery (green) are shown.

The distance between the center point of the AR image of the RLN and that of the actual RLN was measured using the Root Mean Squared Error (RMSE) approach. For the pixel unit to mm unit conversion, the width of the surgical instrument was pre-measured in millimeters and matched to the width of the surgical instrument in pixel units which appeared on the monitor. The measured width of the surgical instrument was 5 mm, and the pixel unit on the screen could be converted into mm unit regardless of camera magnification. Figure 5 demonstrates the procedures of distance measurement using RMSE.

## Supplementary information

Supplementary information.

Supplementary Video S1

## Data Availability

The data that supports the findings are available upon request from the corresponding author.
