# Supplementary material for: Vision-based tracking system for augmented reality to localize recurrent laryngeal nerve during robotic thyroid surgery
Source: Sci Rep. 2020 May 21;10:8437. doi: 10.1038/s41598-020-65439-6 (PMC7242458; doi:10.1038/s41598-020-65439-6)
Supplement: Supplementary file 1 — Supplementary information. [file 41598_2020_65439_MOESM1_ESM.pdf]

**Vision-based tracking system for augmented reality to localize recurrent laryngeal nerve during robotic thyroid surgery**

Dongheon Lee [1,†], Hyeong Won Yu [2,†], Seunglee Kim [3], Jin Yoon [2], Keunchul Lee [2], Young Jun Chai [4,*], June Young Choi [2,*], Hyoun-Joong Kong [5], Kyu Eun Lee [6], Hwan Seong Cho [7], and Hee Chan Kim [8]

## Supplementary Video

**Video S1.** Application of simultaneous localization and mapping technique for augmented reality image overlay during robotic thyroid surgery.
